# My Cat and Me—A Study of Cat Owner Perceptions of Their Bond and Relationship

**DOI:** 10.3390/ani11061601

**Published:** 2021-05-29

**Authors:** Mauro Ines, Claire Ricci-Bonot, Daniel S. Mills

**Affiliations:** Animal Behaviour, Cognition and Welfare Group, School of Life Sciences, University of Lincoln, Lincolnshire LN6 7TS, UK; maurovetbehaviourist@gmail.com (M.I.); dmills@lincoln.ac.uk (D.S.M.)

**Keywords:** affectional bond, attachment, cat, human–animal interaction, owner, relationship, social support, temperament

## Abstract

**Simple Summary:**

Despite the cat’s popularity as a companion animal, little is known about its bond and relationship with owners. The aim of this study was to identify and characterize the different types of relationship that cats might establish with their owners, using human attachment and social support theories as a framework for the underpinning bond. A questionnaire was developed to gather information regarding different emotional elements that could underpin the relationship; the cat’s potential perception of the owner as a secure base; the owner’s level of engagement with the cat, their sensitivity to the cat’s needs and the consistency of their interactions with the cat. Five distinct forms of cat–owner relationship were identified. These seemed to constitute what we describe as an: “open relationship”, “remote association”, “casual relationship”, “co-dependence” and “friendship”. The extent to which these relationships involved a bond towards the owner as a source of social support or secure attachment varied. Accordingly, we conclude that the cat–owner bond should not be profiled simply or solely in terms of attachment in its classic psychological sense.

**Abstract:**

Cats form close emotional relationships with humans, yet little is known about this. This study characterized different types of relationship that cats might establish with their owners. Data were analyzed from 3994 responses to a questionnaire developed using expressions of social support and attachment in relation to everyday cat–owner interactions. Principal component analysis reduced the items to four factors: the “owner’s emotional investment in the cat”, “cat’s acceptance of others”, “cat’s need for owner proximity” and “cat’s aloofness”. Cluster identified three groups of owners with two of these each sub-divided into two. The “open relationship bond” was characterized by a lightly emotionally invested owner and an avoidant cat. The “remote association” and “casual relationship” were characterized by an emotionally remote owner but differed in the cat’s acceptance of others. The “co-dependent” and “friendship” relationship were characterized by an emotionally invested owner but differed in the cat’s acceptance of others and need to maintain owner proximity. In conclusion, as with any complex social relationship, the type of cat–owner bond that develops is the product of the dynamic that exists between both the individuals involved, along with certain personality features, of which, the wider sociability of the cat and owner expectations may be particularly important.

## 1. Introduction

Social support contributes to well-being and has been linked to physical and psychological health outcomes [1]. Animals can form and benefit from partnerships with conspecifics and maintain proximity to others even if not attached or otherwise bonded to them [2,3,4]. Domestication may have enabled humans to assume a role whereby they may provide social support to another species [5,6,7]. A companion animal can also be a source of social support for its owner [8,9]; i.e., the animal’s presence will not only improve the owner’s everyday life but it can also potentially help the owner to cope with stressful situations [1] and thus increase the owner’s resilience [10]. Cats have been reported to be a source of emotional support for some owners [11], especially as a form of non-judgmental confidante [12,13].

Interpersonal bonds describe the reason why individuals are together; at its simplest, it can be divided into physical and psychological bonds. A physical bond exists between a cat and its owner if the cat is not let out, as the cat is physically restricted from leaving the owner. A psychological bond indicates a psychological reason for two individuals being together; this could include a shared purpose, such as working together or a common interest (as occurs with many working teams), but also affectionate emotional bonds. An affectionate bond is recognized from a predisposition to want to be associated with another, which is characterized by its emotional content [2]. There are many forms of emotional bond; for example, the bond between a dependent (care receiver) and its caregiver is different to the bond between the caregiver and its dependent. The former is often characterized by reference to attachment behaviours, whereas the latter is characterized by care-giving or nurturing styles [2]. Thus, bonds are not necessarily reciprocally balanced, and one might exploit the other; the relationship that emerges will reflect this. So, whereas the bond reflects the nature of the interactions of one towards another, the relationship describes the dynamic between the individuals arising from the bonds each have towards the other as well as other potential factors. The relationship between an owner and a companion animal may reflect an enduring tie such as an affectional bond in which the other is emotionally important as a unique individual and is interchangeable with no other [2]. From a historical perspective, attachment is one type of affectional bond, a strong emotional connection that provides security, comfort and the confidence to engage in other activities [2,14], and it is in this context that we use the term “attachment” in this article, in accordance with earlier work that has sought to operationally define the cat–owner relationship (e.g., [15,16]), to distinguish it from other types of emotional bond that might characterize the relationship. Attachment, in this context, is characterized by a desire to maintain closeness to the other (typically as a source of safety and security), and, following separation, pleasure upon reunion. Separation tends to cause distress and persistent loss would cause grief [2]. In species such as the dog, it has been argued that the bond between dog and owner is largely attachment-like in many ways, and this is supported by the behaviour of dogs in the strange situation test; this test operationalizes the definition of attachment for research purposes [17]. This conclusion is supported for the dog, even when a counterbalanced version of the test is used [18], but not in the cat, when a similar test is used [16]. Accordingly, it may be that the bond is different in the cat, and this might reflect the roles they predominantly fulfil. For example, companion animals may also be able to fulfil the reciprocal role of the provider of safety and security in an attachment relationship [19], despite the owner often being referred to as the carer. They can also provide comfort to their owner at times of distress [20], they may be a source of joy and comfort [21,22,23], and they may be missed when absent [22]. These reflect a diversity of emotions and so caution is warranted in emphasizing just the importance of attachment when characterizing the bond and associated relationship. The emotional complexity of the affectionate bond that keeps individuals together needs to be recognized, considering the full scope of the social roles undertaken by the partners at different times (e.g., playmate, caregiver, etc.) and the type of support this may provide.

The use of self-report questionnaires is common practice in attachment and relationship research [19]. There are several scales to measure the quality of the pet–owner relationship [24,25,26,27,28,29,30] but many are not sensitive to species-specific features. However, Howell et al. [30] adapted the Monash dog–owner relationship scale (MONASH) [29] to develop the cat–owner relationship scale (CORS) to assess the quality of the cat–owner relationship; this scale might provide limited insight into the complexity of the owner–cat relationship given its ultimate grounding in the dog literature. Cats are potentially social animals, able to form stable cooperative intraspecific groups but also interspecific relationships with humans and with other domestic species [31]. In Australia, the United Kingdom and the United States of America, about one quarter to one third of households have at least one cat [32,33,34]. However, a high number of cats is relinquished every year [33,35] with behaviour problems and changes in owner circumstances, two of the leading causes of relinquishment [35]. About 77% of owners reported that their cat had at least one unwanted behaviour; a significant number of these related to chronic stress and inadequate resources for them [34], suggesting that owner expectations about the behaviour and needs of the cat may often be poor. About one third to a half of veterinary surgeons are reported to have concerns regarding obesity, access to veterinary care, chronic stress and the provision of inadequate resources to their feline patients [34]. A better understanding of the nature of the relationship that exists between cats and owners could therefore help us provide better care for cats, improve the relationship between cat and owner, and provide greater insight into the potential benefits and limitations of cat ownership [19].

The overall aim of this study was to identify and characterize owner perceptions of the different types of relationships that cats establish with them by using attachment and social support theories as a theoretical foundation for the emotional bonds underpinning them [14,19]. Firstly, we needed to develop a reliable instrument (a questionnaire survey) to explore features of social support, affectionate bonds and attachment (including attachment styles) that can produce affiliation. Secondly, we examined how different items in the instrument relate to each other to form principal components that may be explained by reference to specific underlying psychological constructs. Finally, we could then use scores relating to different components to define different forms of cat–owner relationship and assess their possible association with demographic features, to appreciate the potential nature of the underpinning emotional bonds.

## 2. Materials and Methods

The study was approved by the delegated authority of the University of Lincoln, Research Ethics Committee (reference: CoS 2020-1069).

### 2.1. Questionnaire Creation

A four-part questionnaire was prepared and distributed using an online survey software Qualtrics^XM^ (Supplementary Table S1). The first part consisted of five items related to owner demographic information (age, gender, household size, country of origin and country of residence) and the second part, also with five items, referred to cat demographic information (age, gender, origin, number of cats owned, access to outdoors). Owners were instructed that if they had more than one cat, then they should answer the survey with regards to the cat whose name comes first in the alphabet. The third part consisted of a total of 93 Likert matrix scale items, generated on the basis of their theoretical application to the cat–owner relationship, with eight scale points (from strongly disagree to strongly agree, including a not applicable option). This part was subdivided into the following three subsections:

- *Owner’s caregiving style*—the 38 items in this first subsection reflected owner behaviours and attitudes related to their level of engagement with the cat, their sensitivity to the cat’s needs and their consistency in the interactions with the cat (adapted from the related human literature of the pioneers Bowlby [36] and Ainsworth [2,37].
- *Emotional basis to the relationship between the cat and its owner*—the second subsection, with 25 items, was designed to identify different ways in which the owners might be perceived emotionally by their cats (i.e., different forms of emotionally competent stimuli [38,39]).
- *Traditional attachment features*—the 30 items in the third subsection explored a variety of aspects of cat–owner interactions regarding the owner’s ability to provide social support, the uniqueness of the relationship between the cat and the owner, and the cat’s perception of the owner as a secure base [2,5,37,40,41].

In the last part, participants were asked if they would be willing to participate in another survey. The full survey took about 15 min to complete.

Initially designed in English, the questionnaire was translated to Portuguese by one of the author’s (MI). Two Portuguese native speakers were asked to translate the questionnaire back to English. Their version was compared with the original by another of the authors (DM), to establish consistency of language.

The questionnaire was piloted for comprehensibility, by being sent to 12 cat owners in each language, who were non-scientist cat owners in line with our target population; only minor corrections were made to produce the final version.

### 2.2. Data Collection

The survey was advertised through social media, including personal and group (with permission) Facebook sites, from the 19 July 2020 to 7 September 2020. Social media allows the recruitment of a large population using a snowball sampling method; i.e., within an initial subject group, each subject will in turn refer to new subjects, and so on [42]. This method allows the expansion beyond immediate contacts who may be closely related to the original sources [43]. This method does, however, restrict the control the researcher has over the selection of subjects, introduce a possible bias in so much that the details only reach interconnected individuals, potentially excluding a part of the population [44]. However, in the current case, these are not important issues, since we do not seek to describe the true prevalence of what we find. Inclusion criteria for the study were that participants were over 18 years old and that they complete the questionnaire for only one cat that they have currently lived with for at least six months. They could choose to take the survey in English or Portuguese.

### 2.3. Data Analysis

The data were analyzed using SPSS (v25, IBM).

#### 2.3.1. Item Reliability Assessment

To assess the reliability of items, the questionnaire was sent a second time, via email, approximately one month after their first response, to respondents who had agreed to participate in a second survey. The responses obtained were paired with the corresponding first response to the questionnaire. Since our subsequent analysis focused on the pattern of and relationship between responses in the third part of the questionnaire, reliability was assessed via the correlation and assessment of significant differences in these items between the first and second survey. A Pearson correlation coefficient greater than 0.7 and significant (at *p* < 0.01) was required for retention of an item. Items must also not be significant different, and so those meeting the first requirement were rejected if their value differed significantly between the two surveys (Wilcoxon matched pairs test). Only items meeting these two requirements were retained in the next phase of analysis, as this indicated correlation with no significant shift away from an intercept centred on 0.

#### 2.3.2. Latent Structure of Reliable Items

To investigate how different items in the questionnaire related to each other, a principal component analysis (PCA) was used with the reliable items from the third part of the questionnaire. An oblique rotation (direct oblimin) was used for extraction as it was assumed that the different items may be related [45]. The pattern matrix was used to determine the weight of the different factors in the PCA [45]. The number of principal components (PCs) to extract was decided based on the point of inflexion on the scree plot combined with the Kaiser criterion. For interpretation of the principal components, only the items with a coefficient greater than 0.4 were taken into account.

#### 2.3.3. Population Structure

Principal component scores for each of the respondents were generated. A hierarchical cluster analysis (using Ward’s method and the squared Euclidian distance) was then used to describe the relationship between subjects. The resulting dendrogram was then assessed for viable clusters (population groups).

#### 2.3.4. Group Characteristics

The groups were characterized on the basis of the median scores of each PC. Significant differences between the groups were detected using a Kruskal–Wallis test (since the standardized residuals of the scores of the different PCs in each cluster were not normally distributed). The clusters were also assessed for significant differences in the scores of the items that did not load on any PC using a Kruskal–Wallis test.

Possible significant differences between the clusters related to demographics were assessed with either a Kruskal–Wallis test (owner and cat age) or Chi-squared test (language used to answer the questionnaire, cat and owner gender, household size, owner’s country of origin and residence, cat origin, number of cats owned and access to outdoors). When the Kruskal–Wallis test was used, post hoc tests for pairwise comparisons were used to identify between which groups there were significant differences. When the Chi-squared test had been used, the standardized residuals identified demographic features significantly more or less prevalent in a particular group.

The results were considered significant when *p* < 0.05, adjusted, with a Bonferroni correction applied when multiple tests were performed on related variables.

## 3. Results

There were 6965 responses to the questionnaire. After removing responses with an inappropriate owner or cat age entry (e.g., multiple age entries given), 6357 remained (dataset A).

The majority of the respondents were female (91.7%), who lived (71.1%) and grew up (68.1%) in the United Kingdom in a two-person household (44.8%). Most cats were neutered (94%), had some form of access to outdoors (70.5%) and either lived on their own (40.9%) or shared the house with another cat (32.4%) (Supplementary Table S2).

### 3.1. Item Reliability Assessment

A total of 237 respondents were sent the questionnaire a second time, and 75 responses were received. In order to compare full sets of answers, all the responses that had at least one blank answer were removed from the dataset. This resulted in 56 paired response sets.

A total of 26 of the 93 items composing part three of the questionnaire (9 out of 38 in subsection one, 6 out of 25 in subsection two, 11 out of 30 subsection three) were retained for further analysis (Supplementary Tables S3 and S4).

### 3.2. Latent Structure of Reliable Items

Of the 6357 responses in dataset A, any that had a non-response to one or more of the remaining 26 items were excluded. A total of 3994 remained (dataset B). Dataset B (Table 1) was visually inspected and considered to resemble dataset A, and was thus used in the PCA.

Four PCs were extracted, accounting for 39.4% of the total variance. There were 22 items with a coefficient greater than 0.4 loading on the PCs and four items that did not load on any PC (Table 2 and Supplementary Table S5).

The first PC accounted for 19.5% of the total variance and was composed of 10 items related to the owner’s emotional connection to the cat and was labelled the “owner’s emotional investment in the cat”. The second PC explained 7.3% of the total variance; its four items appeared to be associated with the way the cat related to others and was labelled the “cat’s acceptance of others”. The third PC was composed of five items that appeared to be related to behaviours that involve an effort or reflect a desire to maintain proximity to the owner; it accounted for 6.4% of the total variance and was labelled the “cat’s need for owner proximity”. The three items making up the fourth PC seemed to be related to the cat’s friendliness towards the owner (with a positive score suggesting some degree of intolerance to the owner’s presence or a need for independence); it accounted for 6% of the total variance and was labelled the “cat’s aloofness”. From now on, for narrative purposes, these labels will be used when referring to the specific PCs.

### 3.3. Population Structure

**Figure 1:**
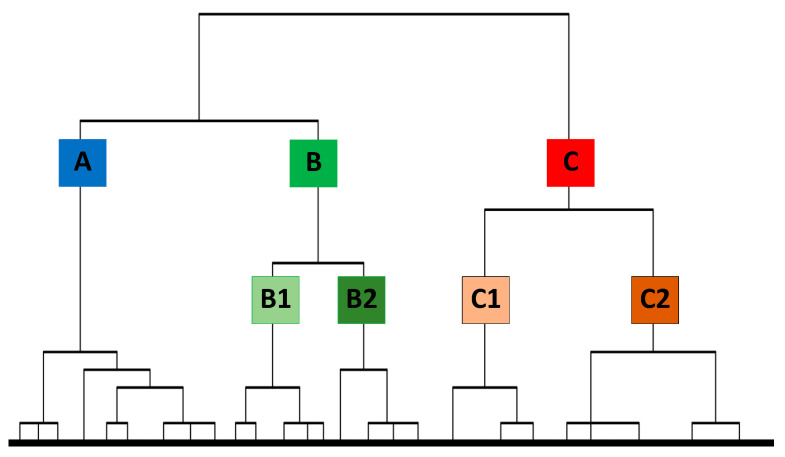
Dendrographic representation of hierarchical cluster analysis of subjects, based on respondents’ score for each PC. The letter represents the different groups.

Three high-level groups: A (N = 992, 28.4%), B (N = 948, 27.1%) and C (N = 1556, 44.5%) emerged from the population (Figure 1), with groups B and C further subdivided into two groups each: B1 (N = 506, 14.5%), B2 (N = 442, 12.6%), C1 (N = 576, 16.5%) and C2 (N = 980, 28%).

### 3.4. Group Characterisitics

The PC scores were significantly different between groups A, B and C (N = 3496, 2 d.f): the “owner’s emotional investment in the cat” (X^2^: 958.621, *p* < 0.001), “cat’s acceptance of others” (X^2^: 225.969, *p* < 0.001), “cat’s need for owner proximity” (X^2^: 584.298, *p* < 0.001) and “cat’s aloofness” (X^2^: 1209.660, *p* < 0.001) (Supplementary Figure S1 and Table S6). Post hoc tests showed that all the groups differed in their scoring of all the PCs (*p* < 0.001).

**Figure 2:**
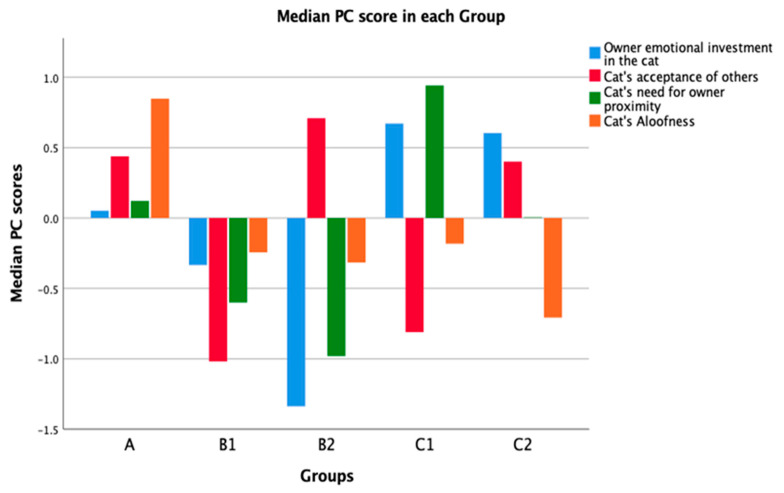
Bar chart representing the median PC scores in groups A, B1, B2, C1 and C2.

The PC scores also differed significantly between groups A, B1, B2, C1 and C2 (N = 3496, 4 d.f, see Figure 2 to help with interpretation of direction of differences).

*“Owner’s emotional investment in the cat”* varied between groups (X^2^: 1011.240, *p* < 0.001). Post hoc tests showed that, with the exception of groups C1 and C2 (*p* = 0.91), all groups differed in their scoring of this PC (*p* < 0.05).
*“Cat’s acceptance of others”* varied between groups (X^2^: 1289.129, *p* < 0.001). Post hoc tests showed that, with the exception of groups A and C2 (*p* = 1.000), all groups differed in their scoring of this PC (*p* < 0.05).
*“Cat’s need for owner proximity”* varied between groups (X^2^: 905.371, *p* < 0.001). Post hoc tests showed that, with the exception of groups A and C2 (*p* = 0.45), all groups differed in their scoring of this PC (*p* < 0.05).
*“Cat’s aloofness”* varied between groups (X2: 1364.305, *p* < 0.001). Post hoc tests showed that, with the exception of groups B1 and B2 (*p* = 1.000), groups B1 and C1 (*p* = 0.167) and groups B2 and C1 (*p* = 1.000), all groups differed in their scoring of this PC (*p* < 0.05).

The median PC scores for groups A, B1, B2, C1 and C2 is represented in Figure 2 (Supplementary Table S7).

There were four reliable items that did not load on any PC. Their scores were significantly different between groups A, B1, B2, C1 and C2.

“I don’t think it is necessary to play with my cat regularly” varied between groups (X^2^: 244.531, *p* < 0.001). Post hoc tests showed that, with the exception of groups A and B1 (*p* = 1.000) and groups C1 and C2 (*p* = 1.000) (Table 3 and Figure S3), all groups differed in their scoring of this item (*p* < 0.05).

“When worried or afraid, my cat will seek me” varied between groups (X^2^: 398.296, *p* < 0.001). Post hoc tests showed that, with the exception of groups B1 and B2 (*p* = 1.000) and groups C1 and C2 (*p* = 1.000) (Table 3 and Figure S4), all groups differed in their scoring of this item (*p* < 0.05).

“My cat will often lick my hands or face” varied between groups (X^2^: 145.274, *p* < 0.001). Post hoc tests showed that, with the exception of groups A and B1 (*p* = 0.620), groups A and B2 (*p* = 1.000), groups B1 and B2 (*p* = 1.000) and groups C1 and C2 (*p* = 1.000) (Table 3 and Figure S5), all groups differed in their scoring of this item (*p* < 0.05).

“When my cat is eating, I like to stay with him/her until he/she has finished” varied between groups (X^2^: 287.364, *p* < 0.001). Post hoc tests for pairwise comparisons showed that, with the exception of groups A and C2 (*p* = 0.649) (Table 3 and Figure S6), all groups differed in their scoring of this item (*p* < 0.05).

The owner’s age was significantly different between groups A, B1, B2, C1 and C2 (N = 3494, X^2^: 79.709, df = 4, *p* < 0.001) (Supplementary Figure S7) and the cat’s age was significantly different between groups A, B1, B2, C1 and C2 (N = 3447, X^2^: 34.996, df = 4, *p* < 0.001) (Supplementary Figure S8).

Other demographic features also varied between groups with the specific effects summarized in Table 4: household size (X^2^: 41.480, df = 12, *p* < 0.001), country the owner lives in (X^2^: 90.974, df = 28, *p* < 0.001), number of cats in the house (X^2^: 69.072, df = 12, *p* < 0.001) and access to outdoors (N = 3496, X^2^: 100.844, df = 4, *p* < 0.001).

No significant differences in group distribution were found relating to: the language used to answer the questionnaire (X^2^: 7.711, df = 4, *p* = 0.103), owner’s gender (X^2^: 22.445, df = 20, *p* = 0.317) and cat’s origin (X^2^: 31.063, df = 20, *p* = 0.054).

## 4. Discussion

This study identified a suite of reliable items that can be used to characterize the cat–owner relationship. These were grouped via principal component analysis largely into four factors: the “owner’s emotional investment in the cat”, “cat’s acceptance of others”, “cat’s need for owner proximity” and “cat’s aloofness”. A further four reliable items (“I don’t think it is necessary to play with my cat regularly” and “when worried or afraid, my cat will seek me” varied between groups; “my cat will often lick my hands or face” and “when my cat is eating, I like to stay with him/her until he/she has finished”) did not associate with any of the principal components. In addition, more than two thirds (67/93) of items were found not to be reliable. This is not surprising given the dynamic nature of our emotional responses, i.e., how we feel emotionally about something may be heavily influenced by recent interactions and cautions against an assumption that such measures are necessarily stable and suitable for use in questionnaire-based studies [46]. There are also suggestions that the behaviour of cats might also be very variable over time in relation to specific events [16], which could further contribute to the unreliability of the items relating to the perception of the cat’s behaviour across time. Although the social relationship between individuals and the associated emotional bonds that define them can change over time, these phenomena are considered relatively stable. Thus, in the current study, the decision to only use items shown to be stable over a one month period excludes transient features that might otherwise be considered important to the relationship. The data from the principal component analysis can be viewed in terms of both what the cat is perceived to do and how this impacts the owners’ feelings about the cat. Thus, from the owner’s perspective, the components could be construed to represent their emotional investment in the cat, the perceived loyalty/faithfulness of their cat to them, or its emotional closeness and enjoyment of mutual physical interaction. However, in terms of the cat’s behaviour, it seems that these perceptions might arise from the degree to which the cat’s responses are compatible with the owner’s needs, (perhaps akin to the social sensitivity that is widely recognized as important in our relationship with dogs [47]), the gregariousness of the cat (a commonly recognized personality trait of many species [48]), the ability to cope with the owner’s absence (a trait which might relate more to frustration tolerance rather than attachment [16] and tolerance of physical contact (a highly variable feature between and within cats [49,50]). When combined with the other specific reliable items relating to the perception of play—licking of the owner, tolerance of the owner when eating and seeking out the owner when afraid—it seems that these features might provide important underpinnings for both the type of relationship that develops and also owner satisfaction. It is noticeable that the seeking of the owner when worried did not converge with other items that might suggest traditional attachment and we would therefore suggest, as reported previously [16], that the bond between the cat and its owner is not well-characterized by reference to this concept as the sole or primary feature defining it. Our data suggest that the relationship that emerges from the complex interaction of the behaviour of the cat and emotional needs of the owner can be constructed hierarchically with three high-level groups at its simplest but is perhaps best-represented by five type of relationship, which we discuss further below.

The first top-level group and only one not to clearly subdivide seemed to represent a population with an “*open relationship*” (28.4% of the population). It is characterized by a neutral or balanced level of emotional investment in the cat. The cats typically have access to outdoors, relate well to other people but also have some affiliation with the owner, but have little need for owner proximity and may be seen as aloof or independent. This is perhaps the prototypical view of the nature of the cats as largely solitary, independent animals (these owners are less likely to own large numbers of cats) and associated cat–owner relationship [51]. Therefore, in the five-group model, it is not surprising that this is one of the largest groups, representing about a quarter of owners in our sample. While we describe this as an open relationship, it should be noted that this does not mean the owner does not care for the cat; indeed, they probably recognize the importance of enrichment, as evidenced by their tendency to allow the cat outdoors [52], and that it is quite important to play with the cat. However, the role of the owner as any form of secure base seems to be relatively weak given the response to the item on the tendency to seek out the owner when distressed.

The second top level group represented 27.1% of the population and was characterized by a low level (negative score) of owner emotional investment in the cat. This would suggest that the cat is not considered a friend or a part of the family and perhaps the strength of affiliation between the owner and cat may be low, even though the cat’s behaviour towards the owner may be quite friendly (negative score on the “cat’s aloofness” component). However, the cat does not appear to try to maintain proximity from the owner (low score on “cat’s need for owner proximity”), who does not seem to function as a secure base. The two subgroups within this type of relationship appear to differentiate largely on the basis of the cat’s sociability/acceptance of others, alongside the owner’s emotional investment. Those in group B1 score negatively on the cat’s acceptance of others and slightly negatively on the owner’s emotional investment; accordingly, we refer to this as a “*remote association*”, since the cat does not appear to express many affiliative tendencies (low scores for the “cat’s acceptance of others” and “cat’s need for owner proximity”). The low positive score on the “cat’s aloofness” combined with the fact that the score on the “cat’s acceptance of others” seems more negative than the score on the “cat’s need for owner proximity” might suggest that the cat tolerates the owner more than others, but there are no strong signs of affiliation or use of the owner as a secure base. By contrast, we describe B2 as having a “*casual relationship*”, since the cat is clearly sociable and accepting of others but has the least need for owner proximity of any of the groups. Thus, there is little evidence that the cat discriminates the owner from others. It is perhaps not surprising, then, that these casual relationships typically occur in busy households. The cats have access to outdoors and given that they have the highest score on the “cat’s acceptance of others”, it would not be surprising if these cats are the type that visit other households in their vicinity, which can be problematic [53].

The third top level group (44.5% of the population) is characterized by the highest positive score in the “owner’s emotional investment in the cat”, low levels of aloofness alongside a high score for the specific item “my cat will often lick my hands or face”, which is widely recognized as a sign of social affiliation [31]. Thus, this group appear to be an affectionate group of cats. Superficially, it seems that this group of cats has a general need for owner proximity. However, closer inspection of the subgroups suggests that this is an oversimplification, as the population appears to divide on the basis of this trait, into those with a high score and those with a more neutral score. The division is also marked by contrasting differences in the cat’s acceptance of others, being very low among those who seek owner proximity more, perhaps suggesting a more exclusive relationship between cat and owner in this case. Given the high owner investment in this sub-group, we refer to the relationship as one of co-dependence. The “*co-dependent*” relationship appears to typically occur in a one-person household, in Brazil, Mexico, South Africa or the United States, with a cat that does not have access to outdoors. The cat and owner will often play together, and the owner is likely to stay with the cat whilst the cat is eating. This suggests that the relationship might be largely conditioned from owner interaction, in the same way that some cat vocalizations might be [54]. Given this profile, it would be useful to determine whether this group are at higher risk of separation related problems [55], which has been hypothesized to relate to a close attachment bond [55] and/or poor frustration tolerance of reduced owner reinforcement and provision of resources [16].

It is worth noting that the general sociability of the cat appears to be important again in determining the type of relationship that develops with the owner within this third top-level group, with the second subgroup being generally more accepting of others, but also less aloof. We refer to this relationship as a “*friendship*”. This type of relationship occurs more often in houses with more than one cat (and so there is less exclusive association between the owner and their cat). The neutral score relating to the cat’s need for owner proximity and the fact that the owner is less likely to stay with the cat whilst the cat is eating (compared to the “co-dependent” relationship) suggests that, in spite of having an apparently friendly and warm relationship, the cat and owner can function independently. This cat likes to be near the owner but does not feel a need to maintain physical proximity to the owner.

A limitation of this study is that the sample is self-selected. In fact, the majority of participants were females living in the United Kingdom (66.2% of the population). Thus, the proportions of the population falling into each category cannot be assumed to represent those found in the wider cat-owning demographic, nor can we exclude the potential for other types of relationship. We also recognise that this study is based on the owner’s perception of the relationship and many of the inferences we make about the underlying mechanism remain speculation. Nonetheless, we suggest that it is important to move beyond a model of the relationship focused only on attachment [56]. Our embracing of the emotional complexity of cat owner relationships provides a solid foundation for future work relating to both owner satisfaction and the development of problem behaviours. Future work should investigate our results further with more controlled studies utilizing more objective measures of the bond. We suggest that the potential role of owner reinforcement on the shaping of the cat’s relationship is an area of particular importance, given the demographic associations found here.

## 5. Conclusions

Our data indicate that the perceptions of cat owners describe five distinct types of relationship (see Information S1 for final English and Portuguese versions of the instruments developed). The owner’s level of emotional investment in the cat and the cat’s sociability appear to be particularly important in discriminating these. Sociability in terms of acceptance of others should not be confused with the close contact and exchanges associated with affiliation, and while many cats may be aloof, it seems that this is not as common as might be commonly portrayed.

## Figures and Tables

**Table 1:** Dataset B—population distribution by demographic category, after removing from the dataset all the responses that had at least one blank answer to the 26 items considered reliable. All variables were within 1% of dataset A, except: household size—2 (1.1%), cat gender—male neutered (1.2%), number of cats—1 (1.2%) and 2 (1.2%).

Demographic		Dataset B (3994 Responses)
Language	English	3877 (97.1%)
	Portuguese	117 (2.9%)
Owner age (mean, standard deviation, min–max)		41.49 +/− 12.75 (18 to 83 years)
Owner gender	Female	3662 (91.7%)
	Male	258 (6.5%)
	Transgender female	0 (0%)
	Transgender male	9 (0.2%)
	Gender variant/non-conforming	42 (1.1%)
	Not listed	15 (0.12%)
	Prefer not to answer	8 (0.2%)
Household size	1	694 (17.4%)
	2	1834 (45.9%)
	3 or 4	1250 (31.3%)
	>4	216 (5.4%)
Country of residence	United Kingdom	2867 (71.7%)
	United States	399 (10%)
	Ireland	130 (3.3%)
	South Africa	99 (2.5%)
	Australia	97 (2.5%)
	Portugal	91 (2.3%)
	Canada	45 (1.2%)
	Other	266 (6.5%)
Country grew up in	United Kingdom	2750 (68.8%)
	United States	422 (10.6%)
	South Africa	113 (2.9%)
	Ireland	104 (2.6%)
	Portugal	98 (2.5%)
	Australia	81 (2%)
	Canada	62 (1.6%)
	Other	364 (9%)
Cat age (mean standard deviation, min–max)		7.17 +/− 4.63 (0 to 25 years)
Cat gender	Male entire	91 (2.3%)
	Female entire	108 (2.7%)
	Male neutered	2006 (50.2%)
	Female neutered	1788 (44.8%)
	Not sure	1 (0%)
Cat origin	Breeder	397 (10%)
	Shelter/charity	1547 (38.7%)
	Friend	743 (18.6%)
	Petshop	36 (0.9%)
	Advert	411 (10.3%)
	Other	860 (21.5%)
Number of cats	1	1586 (39.7%)
	2	1345 (33.6%)
	3	480 (12%)
	4 or more	583 (14.6%)
Access to outdoors	Yes	2835 (71%)
	No	1159 (29%)

**Table 2:** List of reliable items, with the respective loading scores on each PC. The items that loaded onto each individual PC are shown in bold.

Items	PC 1	PC 2	PC 3	PC 4
	Owner’s Emotional Investment in the Cat	Cat’s Acceptance of Others	Cat’s Need for Owner Proximity	Cat’s Aloofness
I don’t know what I would do without my cat.	**0.780**	0.013	0.039	0.046
My cat is my best friend.	**0.744**	−0.036	0.099	0.004
I think of my cat as like a child.	**0.656**	0.014	0.127	0.151
I am very protective of my cat.	**0.584**	−0.057	−0.037	−0.023
I worry too much about my cat.	**0.579**	−0.094	0.125	0.256
If I were to tell my cat off and he/she would start purring or meowing at me, I would stop and try to make up with him/her.	**0.469**	−0.019	0.015	0.164
I am comfortable with being emotionally distant from my cat.	**−0.430**	0.100	0.006	0.309
I consider my cat part of the family.	**0.422**	0.051	−0.134	−0.032
I talk to my cat every day.	**0.412**	0.074	−0.047	−0.077
My cat and I often seem to work as a team that are in tune with each other.	**0.409**	0.077	0.221	−0.319
I don’t think it is necessary to play with my cat regularly.	−0.301	0.008	−0.088	0.198
When I go away my cat will play with and enjoy whoever is looking after him/her.	0.030	**0.756**	−0.062	−0.093
My cat tends to approach visitors with his/her tail up and rub him/herself on their legs.	0.063	**0.732**	0.118	−0.054
My cat visits the neighbours (even if I am at home).	−0.045	**0.540**	−0.006	0.335
I think my cat would be just as happy living with someone else (such as the next-door neighbour).	−0.344	**0.470**	−0.056	0.376
My cat constantly follows me around the house.	−0.015	−0.002	**0.765**	−0.108
My cat is clingy. I can’t even go to the toilet in peace.	−0.011	−0.011	**0.716**	−0.111
If I am in a separate room with a closed door, my cat will cry until I open the door.	0.046	−0.005	**0.683**	−0.002
When I am about to leave the house, my cat will cry and try to leave with me.	−0.034	0.083	**0.661**	0.120
My cat will not eat if I go away.	−0.068	−0.317	**0.455**	0.133
When worried or afraid, my cat will seek me.	0.259	0.147	0.323	−0.249
My cat will often lick my hands or face.	0.042	0.140	0.323	−0.215
When my cat is eating, I like to stay with him/her until he/she has finished.	0.241	−0.119	0.297	0.166
I feel my cat doesn’t like me as much as like him/her.	−0.037	0.054	−0.116	**0.700**
My cat will never sit on my lap.	0.046	−0.172	−0.136	**0.520**
When siting on my lap, being fussed, my cat’s tail will sometimes thrash.	0.084	0.074	0.135	**0.433**

**Table 3:** Median rating on the items not loaded on any PC, in each of the groups. Note that contrasts between clusters are all significant, except where indicated.

Item (Median Answer on the Questionnaire)	Group				
	A	B1	B2	C1	C2
I don’t think it is necessary to play with my cat regularly	Mainly disagree	Mainly disagree	Mainly disagree	Strongly disagree	Strongly disagree
	(Not significantly different from B1)	(Not significantly different from A)		(Not significantly different from C2)	(Not significantly different from C1)
When worried or afraid, my cat will seek me	Partly agree	Partly agree	Partly agree	Mainly agree	Mainly agree
		(Not significantly different from B2)	(Not significantly different from B1)	(Not significantly different from C2)	(Not significantly different from C1)
My cat will often lick my hands or face	Partly agree	Partly agree	Partly agree	Mainly agree	Mainly agree
	(Not significantly different from B1 and B2)	(Not significantly different from A and B2)	(Not significantly different from A and B1)	(Not significantly different from C2)	(Not significantly different from C1)
When my cat is eating, I like to stay with him/her until he/she has finished	Mainly disagree	Strongly disagree	Strongly disagree	Partly disagree	Mainly disagree
	(Not significantly different from C2)				(Not significantly different from A)

**Table 4:** Demographic features, less and more prevalent in the different groups (z = standardized residuals).

Group	Less Prevalent	More Prevalent
A	Owner lives in the United States (z = −2.7) If four or more cats live in the house (z = −2.9) Cat has no access to outdoors (z = −3.7)	If only one cat lives in the house (z = 2.7) Cat has access to outdoors (z = 2.0)
B1	If four or more cats live in the house (z = −2.1)	
B2	If two people live in the household (z = −2.6) Owner lives in the United States (z = −2.8) Owner lives in South Africa (z = −2.6) Cat has no access to outdoors (z = −4.0)	If three or four people live in the household (z = 3.7) Cat has access to outdoors (z = 2.1)
C1	Owner lives in the United Kingdom (z = −3.3) Cat has access to outdoors (z = −3.7)	If only one person lives in the household (z = 2.0) Owner lives in Brazil (z = 2.5) Owner lives in Mexico (z = 2.4) Owner lives in South Africa (z = 2.4) Owner lives in the United States (z = 4.7) Cat has no access to outdoors (z = 6.9)
C2	If only one cat in the house (z = −3.2)	If four or more cats live in the house (z = 4.3)

## Data Availability

The anonymized data will be held by the first author, and will be made available to others upon reasonable request.

## Supplementary Materials

The following are available online at https://www.mdpi.com/article/10.3390/ani11061601/s1, Figure S1: median PC scores for groups A, B and C; Figure S2: scree plot of PCA of relationship items, four factors chosen before the inflexion point; Figure S3: box plot representing the score for groups A, B1, B2, C1 and C2 for the item: “I don’t think it is necessary to play with my cat regularly”; Figure S4: box plot representing the score for groups A, B1, B2, C1 and C2 for the item: “when worried or afraid, my cat will seek me”; Figure S5: box plot representing the score for groups A, B1, B2, C1 and C2 for the item: “my cat will often lick my hands or face”; Figure S6: box plot representing the score for groups A, B1, B2, C1 and C2 for the item: “when my cat is eating, I like to stay with him/her until he/she has finished”; Figure S7: box plot representing the owner age distribution in groups A, B1, B2, C1 and C2; Figure S8: box plot representing the cat age distribution in groups A, B1, B2, C1 and C2; Table S1: questions in parts 1, 2 and 3 of the survey as they were asked to the participants in the study; Table S2: table comparison of dataset A (responses that remained after removing all the blank responses and all the responses with inappropriate cat and owner age entries) with dataset B (responses that remained after removing all the responses that had at least one blank answer to one of the 26 reliable items in the questionnaire) in all the owner and cat demographics; Table S3: details of the reliability assessment. Results of the Pearson correlation, results of the Wilcoxon test and outcome for each of the 93 items in part 3 of the questionnaire survey; Table S4: The 26 reliable items retained for analysis; Table S5: principal component analysis, total variance explained by all components. All retained components had an eigenvalue greater than 1 (up to 5 component solution possible, based on the Kaiser criterion). The scree plot (S6) was then used to confirm whether a four- or five-factor solution should be used; Table S6: the median PC score for the groups A, B and C; Table S7: the median PC score for the groups A, B1, B2, C1 and C2; Information S1: English and Portuguese versions of final instruments and their relationship to the clusters identified in simple language terms.
