# Supplementary material for: My Cat and Me—A Study of Cat Owner Perceptions of Their Bond and Relationship
Source: Animals (Basel). 2021 May 29;11(6):1601. doi: 10.3390/ani11061601 (PMC8228645; doi:10.3390/ani11061601)
Supplement: Supplementary file 1 [file animals-11-01601-s001.zip › animals-1229120-supplementary.pdf]

Supplementary materials

Table S1: Questions in parts 1, 2 and 3 of the survey as they were asked to the participants in the study - note for conciseness formatting changes have been made and response options removed from Parts 1 and 2.

**PART 1 – Owner demographic information**								
What is your age (to the nearest complete year)?								
With which gender do you most closely identify?								
Number of people in your household?								
In which country do you currently reside?								
In which country did you grow up in?								
**PART 2 – Cat demographic information**								
What is your cat's age (in years)?								
What is your cat's gender?								
Where did you get your cat from?								
How many cats do you have?								
Does your cat have any access outdoors?								
**PART 3, Subsection 1 – Owner's caregiving style**								
Item	Likert scale options							
	Strongly disagree	Mainly disagree	Partly disagree	Neutral/mixed	Partly agree	Mainly agree	Strongly agree	Not appli-cable
I encourage my cat to sleep with me on my bed								
I feel my cat doesn't like me as much as like him/her								
I am comfortable with being emotionally distant from my cat								
I would feel sad if I thought that my cat liked others more than me								
When my cat is eating I like to stay with him/her until he/she has finished								
I consider my cat part of the family								
I am not able to understand what my cat needs								
I don't think it is necessary to play with my cat regularly								
I get upset with my cat if he/she seeks attention when I am busy								
I shout at my cat when he/she does something wrong								
I expect my cat to follow my house rules								
I provide food, water and shelter. My cat should be able to sort everything else he/she needs.								
I would cancel my plans in order to avoid leaving my cat alone								
Sometimes I allow my cat to do things that I don't allow at other times								
I enjoy playing with my cat								
Sometimes I start playing with my cat but then decide against it and so stop playing quite abruptly.								
I think it is difficult to teach my cat what I want him/her to do								
I am comfortable with the relationship I have with my cat								
Sometimes I feel I am not a good enough owner								
I am very protective of my cat								
I make sure my cat gives me attention whenever I want it								
I don't think my cat needs me much								
I enjoy doing all the things related to caring for my cat (i.e feeding, cleaning litter box, playing, etc)								
I talk to my cat everyday								
I don't have strong feelings for my cat								
I recognise when my cat needs help								
I always follow the vet's instructions for the health of my cat								
I worry to much about my cat								
I am often impatient with my cat								
If I were to tell my cat off and he/she would start purring or meowing at me, I would stop and try to make up with him/her								

I'm interested in learning things that will help me take better care of my cat								
When my cat does something wrong, I feel that he/she did that to upset me								
When I am upset with my cat, I stay away from him/her								
I don't want my cat to make mistakes and so I step in whenever I think he/she is making a bad decision								
My cat is very independent. I barely notice he/she lives at home with me								
I often feel the need to support my cat through stressful situations								
My cat has a unique personality								
I don't think it is necessary to reward my cat for doing things he/she knows he/she should be doing								
**PART 3, Subsection 2 – Emotional basis to the relationship between the cat and its owner**								
Item	Likert Scale options							
	Strongly disagree	Mainly disagree	Partly disagree	Neutral/mixed	Partly agree	Mainly agree	Strongly agree	Not applicable
My cat treats me like a toy								
My cat thinks of me as a food dispensing machine								
There aren't enough hours in the day to give my cat all the attention he/she would like								
I deliberately don't let my cat get all that he/she wants								
Sometimes my cat needs some space								
When siting on my lap, being fussed, my cat's tail will sometimes thrash								
My cat gets angry if I don't give him what he/she wants								
I think my cat is afraid of me								
my cat sometimes runs away from me or suddenly hisses or swipes at me for no good reason								
My cat will often hide from me								
I think my cat worries I would hurt him/her								
Sometimes my cat seems irritable or doesn't like to be touched for no obvious reason.								
Sometimes I have to make my cat uncomfortable to get certain things done (e.g. giving medication)								
I think of my cat as like a child								
When worried or afraid, my cat will seek me								
My cat is always there for me, when I am lonely								
I share my worries with my cat								
I don't know what I would do without my cat								
My cat is my best friend								
I wouldn't worry if another person took care of my cat								
My cat loves rough and tumble play								
My cat and I often seem to work as a team that are in tune with each other								
Sometimes my cat shows sexual behaviour (i.e. mounting) towards me								
My cat does not want me to be a part of his/her social circle								
My cat will often try to make my partner or others close to me move away								
**PART 3, Subsection 3 – Traditional Attachment features**								
Item	Likert Scale options							
	Strongly disagree	Mainly disagree	Partly disagree	Neutral/mixed	Partly agree	Mainly agree	Strongly agree	Not applicable
My cat is confident								
When I go away my cat will play with and enjoy whoever is looking after him/her								
My cat will not eat if I go away								
My cat constantly follows me around the house								
If I am in a separate room with a closed door, my cat will cry until I open the door								
My cat will only relax if next to me								
My cat tends to approach visitors with his/her tail up and rub him/herself on their legs								
My cat will never sit on my lap								
When we are both at home, I might not see my cat for the entire day								
When I return home my cat seems annoyed and might even scratch/bite/hiss at me								
When I return home my cat will approach me with his/her tail up and rub him/herself on my legs								
My cat wants to be close to me but will scratch/bite me if I try to touch him/her								
My cat will often lick my hands or face								

When I return home, my cat follows me around and cries, even if I give him/her attention
If I leave the house, I believe my cat would be fine with someone else taking my place
My cat sees our relationship as something special
For my cat attention is attention, whoever gives it, it doesn't matter who it comes from
When I return home my cat ignores me
When I am about to leave the house, my cat will cry and try to leave with me
My cat will sit near whoever has the warmest or most comfortable spot
My cat visits the neighbours (even if I am at home)
If we have visitors my cat will initially sit next to me, and may or may not approach the visitors
My cat will groom him/herself when I arrive home
I think my cat would be just as happy living with someone else (like the next door neighbour)
My cat likes all people equally
My cat seems to know exactly how I feel and acts accordingly
I can never tell how my cat is going to react when I approach him/her
If my cat was anxious, people other than me, might be just as good at comforting my cat
My cat appears aloof
My cat is clingy. I can't even go to the toilet in peace.

Table S2 - Table comparison of Dataset A (responses that remained after removing all the blank responses and all the responses with inappropriate cat and owner age entries) with Dataset B (responses that remained after removing all the responses that had at least one blank answer to one of the 26 reliable items in the questionnaire) in all the Owner and Cat Demographics.

Demographic		Dataset A (6357 responses)	Dataset B (3994 responses)
Language	English	6137 (96.5%)	3877 (97.1%)
	Portuguese	220 (3.5%)	117 (2.9%)
Owner age (mean, standard deviation, min - max)		41.81 +/- 12.98 (18y – 85y)	41.49 +/- 12.75 (18y – 83y)
Owner gender	Female	5826 (91.7%)	3662 (91.7%)
	Male	405 (6.4%)	258 (6.5%)
	Transgender female	5 (0.1%)	0 (0%)
	Transgender male	14 (0.2%)	9 (0.2%)
	Gender variant/non-conforming	63 (1%)	42 (1.1%)
	Not listed	23 (0.4%)	15 (0.12%)
	Prefer not to answer	24 (0.3%)	8 (0.2%)
Household size	1	1107 (17.5%)	694 (17.4%)
	2	2850 (44.8%)	1834 (45.9%)
	3 or 4	2037 (32.1%)	1250 (31.3%)
	> 4	363 (5.7%)	216 (5.4%)
Country of residence	United Kingdom	4523 (71.1%)	2867 (71.7%)
	United States	644 (10.2%)	399 (10%)
	Ireland	212 (3.3%)	130 (3.3%)
	South Africa	135 (2.2%)	99 (2.5%)
	Australia	151 (2.4%)	97 (2.5%)
	Portugal	158 (2.5%)	91 (2.3%)
	Canada	67 (1.1%)	45 (1.2%)
	Other	467 (7.2%)	266 (6.5%)
Country grew up in	United Kingdom	4331 (68.1%)	2750 (68.8%)
	United States	671 (10.6%)	422 (10.6%)
	South Africa	172 (2.7%)	113 (2.9%)
	Ireland	159 (2.5%)	104 (2.6%)
	Portugal	130 (2%)	98 (2.5%)
	Australia	169 (2.7%)	81 (2%)
	Canada	94 (1.5%)	62 (1.6%)

	Other	631 (9.9%)	364 (9%)
Cat age (mean standard deviation, min - max)		7.07 +/- 4.65 (0 – 25y)	7.17 +/- 4.63 (0 – 25y)
Cat gender	Male entire	165 (2.6%)	91 (2.3%)
	Female entire	203 (3.2%)	108 (2.7%)
	Male neutered	3113 (49%)	2006 (50.2%)
	Female neutered	2872 (45%)	1788 (44.8%)
	Not sure	4 (0.1%)	1 (0%)
Cat origin	Breeder	612 (9.6%)	397 (10%)
	Shelter/charity	2461 (38.7%)	1547 (38.7%)
	Friend	1197 (18.9%)	743 (18.6%)
	Petshop	63 (1%)	36 (0.9%)
	Advert	623 (9.8%)	411 (10.3%)
	Other	1401 (22%)	860 (21.5%)
Number of cats	1	2603 (40.9%)	1586 (39.7%)
	2	2062 (32.4%)	1345 (33.6%)
	3	774 (12.2%)	480 (12%)
	4 or more	918 (14.5%)	583 (14.6%)
Access outdoors	Yes	4484 (70.5%)	2835 (71%)
	No	1873 (29.5%)	1159 (29%)

Table S3 - Details of the reliability assessment. Results of the Pearson correlation, results of the Wilcoxon test and outcome for each of the 93 items in Part 3 of the questionnaire survey.

**Item**	**Pearson correlation**		**Wilcoxon test**	**Outcome**
	**Correlation statistically significant (p < 0.05)**	**Correlation coefficient**	**2 answers significantly different (p < 0.05)**	
**Part 3, Subsection 1 – Owner caregiving style**				
**I encourage my cat to sleep with me on my bed**	yes	0.652	no	Removed from dataset.
**I feel my cat doesn't like me as much as like him/her**	yes	0.772	no	Remained in dataset. Used for analysis.
**I am comfortable with being emotionally distant from my cat**	yes	0.722	no	Remained in dataset. Used for analysis.
**I would feel sad if I thought that my cat liked others more than me**	yes	0.663	no	Removed from dataset.
**When my cat is eating I like to stay with him/her until he/she has finished**	yes	0.813	no	Remained in dataset. Used for analysis.
**I consider my cat part of the family**	yes	0.758	no	Remained in dataset. Used for analysis.
**I am not able to understand what my cat needs**	no	0.063	no	Removed from dataset.
**I don't think it is necessary to play with my cat regularly**	yes	0.718	no	Remained in dataset. Used for analysis.
**I get upset with my cat if he/she seeks attention when I am busy**	yes	0.467	no	Removed from dataset.

**I shout at my cat when he/she does something wrong**	yes	0.508	no	Removed from dataset.
**I expect my cat to follow my house rules**	yes	0.577	yes	Removed from dataset.
**I provide food, water and shelter. My cat should be able to sort everything else he/she needs.**	yes	0.593	no	Removed from dataset.
**I would cancel my plans in order to avoid leaving my cat alone**	yes	0.656	no	Removed from dataset.
**Sometimes I allow my cat to do things that I don't allow at other times**	yes	0.350	no	Removed from dataset.
**I enjoy playing with my cat**	no	0.362	no	Removed from dataset.
**Sometimes I start playing with my cat but then decide against it and so stop playing quite abruptly.**	yes	0.529	no	Removed from dataset.
**I think it is difficult to teach my cat what I want him/her to do**	yes	0.534	no	Removed from dataset.
**I am comfortable with the relationship I have with my cat**	no	0.085	no	Removed from dataset.
**Sometimes I feel I am not a good enough owner**	yes	0.586	no	Removed from dataset.
**I am very protective of my cat**	yes	0.784	no	Remained in dataset. Used for analysis.
**I make sure my cat gives me attention whenever I want it**	yes	0.419	no	Removed from dataset.
**I don't think my cat needs me much**	yes	0.595	no	Removed from dataset.
**I enjoy doing all the things related to caring for my cat (i.e feeding, cleaning litter box, playing, etc)**	yes	0.553	no	Removed from dataset.
**I talk to my cat everyday**	yes	0.718	no	Remained in dataset. Used for analysis.
**I don't have strong feelings for my cat**	yes	0.330	no	Removed from dataset.
**I recognise when my cat needs help**	no	0.237	no	Removed from dataset.
**I always follow the vet's instructions for the health of my cat**	no	0.188	no	Removed from dataset.
**I worry to much about my cat**	yes	0.704	no	Remained in dataset. Used for analysis.
**I am often impatient with my cat**	yes	0.576	no	Removed from dataset.
**If I were to tell my cat off and he/she would start purring or meowing at me, I would stop and try to make up with him/her**	yes	0.703	no	Remained in dataset. Used for analysis.
**I'm interested in learning things that will help me take better care of my cat**	yes	0.628	no	Removed from dataset.

**When my cat does something wrong, I feel that he/she did that to upset me**	yes	0.652	no	Removed from dataset.
**When I am upset with my cat, I stay away from him/her**	yes	0.638	no	Removed from dataset.
**I don't want my cat to make mistakes and so I step in whenever I think he/she is making a bad decision**	yes	0.576	no	Removed from dataset.
**My cat is very independent. I barely notice he/she lives at home with me**	yes	0.627	yes	Removed from dataset.
**I often feel the need to support my cat through stressful situations**	yes	0.697	no	Removed from dataset.
**My cat has a unique personality**	yes	0.627	no	Removed from dataset.
**I don't think it is necessary to reward my cat for doing things he/she knows he/she should be doing**	yes	0.560	no	Removed from dataset.
Subsection balance (items retained/total items in Subsection 1)				*9 / 38*
**Part 3, Subsection 2 – Emotional framework**				
**My cat treats me like a toy**	yes	0.441	no	Removed from dataset.
**My cat thinks of me as a food dispensing machine**	yes	0.586	no	Removed from dataset.
**There aren't enough hours in the day to give my cat all the attention he/she would like**	yes	0.560	no	Removed from dataset.
**I deliberately don't let my cat get all that he/she wants**	yes	0.545	no	Removed from dataset.
**Sometimes my cat needs some space**	yes	0.729	yes	Removed from dataset.
**When siting on my lap, being fussed, my cat's tail will sometimes thrash**	yes	0.835	no	Remained in dataset. Used for analysis.
**My cat gets angry if I don't give him what he/she wants**	yes	0.627	no	Removed from dataset.
**I think my cat is afraid of me**	no	0.261	no	Removed from dataset.
**my cat sometimes runs away from me or suddenly hisses or swipes at me for no good reason**	yes	0.529	no	Removed from dataset.
**My cat will often hide from me**	yes	0.453	no	Removed from dataset.
**I think my cat worries I would hurt him/her**	yes	0.270	no	Removed from dataset.
**Sometimes my cat seems irritable or doesn't like to be touched for no obvious reason.**	Yes	0.349	no	Removed from dataset.
**Sometimes I have to make my cat uncomfortable to get certain things done (e.g. giving medication)**	yes	0.619	no	Removed from dataset.

**I think of my cat as like a child**	yes	0.890	no	Remained in dataset. Used for analysis.
**When worried or afraid, my cat will seek me**	yes	0.708	no	Remained in dataset. Used for analysis.
**My cat is always there for me, when I am lonely**	yes	0.746	yes	Removed from dataset.
**I share my worries with my cat**	yes	0.673	no	Removed from dataset.
**I don't know what I would do without my cat**	yes	0.724	no	Remained in dataset. Used for analysis.
**My cat is my best friend**	yes	0.794	no	Remained in dataset. Used for analysis.
**I wouldn't worry if another person took care of my cat**	yes	0.293	no	Removed from dataset.
**My cat loves rough and tumble play**	yes	0.650	no	Removed from dataset.
**My cat and I often seem to work as a team that are in tune with each other**	yes	0.766	no	Remained in dataset. Used for analysis.
**Sometimes my cat shows sexual behaviour (i.e. mounting) towards me**	yes	0.416	yes	Removed from dataset.
**My cat does not want me to be a part of his/her social circle**	no	0.030	no	Removed from dataset.
**My cat will often try to make my partner or others close to me move away**	yes	0.699	no	Removed from dataset.
Subsection balance (items retained/total items in Subsection 2)				***6/25***
**Part 3, Subsection 3 – Cat-owner bond**				
**My cat is confident**	yes	0.699	no	Removed from dataset.
**When I go away my cat will play with and enjoy whoever is looking after him/her**	yes	0.731	no	Remained in dataset. Used for analysis.
**My cat will not eat if I go away**	yes	0.782	no	Remained in dataset. Used for analysis.
**My cat constantly follows me around the house**	yes	0.726	no	Remained in dataset. Used for analysis.
**If I am in a separate room with a closed door, my cat will cry until I open the door**	yes	0.721	no	Remained in dataset. Used for analysis.
**My cat will only relax if next to me**	yes	0.689	no	Removed from dataset.
**My cat tends to approach visitors with his/her tail up and rub him/herself on their legs**	yes	0.741	no	Remained in dataset. Used for analysis.
**My cat will never sit on my lap**	yes	0.818	no	Remained in dataset. Used for analysis.

**When we are both at home, I might not see my cat for the entire day**	yes	0.476	no	Removed from dataset.
**When I return home my cat seems annoyed and might even scratch/bite/hiss at me**	yes	0.289	no	Removed from dataset.
**When I return home my cat will approach me with his/her tail up and rub him/herself on my legs**	yes	0.597	no	Removed from dataset.
**My cat wants to be close to me but will scratch/bite me if I try to touch him/her**	yes	0.669	no	Removed from dataset.
**My cat will often lick my hands or face**	yes	0.780	no	Remained in dataset. Used for analysis.
**When I return home, my cat follows me around and cries, even if I give him/her attention**	yes	0.427	no	Removed from dataset.
**If I leave the house, I believe my cat would be fine with someone else taking my place**	yes	0.550	yes	Removed from dataset.
**My cat sees our relationship as something special**	yes	0.677	no	Removed from dataset.
**For my cat attention is attention, whoever gives it, it doesn't matter who it comes from**	yes	0.614	no	Removed from dataset.
**When I return home my cat ignores me**	yes	0.697	no	Removed from dataset.
**When I am about to leave the house, my cat will cry and try to leave with me**	yes	0.707	no	Remained in dataset. Used for analysis.
**My cat will sit near whoever has the warmest or most comfortable spot**	yes	0.684	no	Removed from dataset.
**My cat visits the neighbours (even if I am at home)**	yes	0.805	no	Remained in dataset. Used for analysis.
**If we have visitors my cat will initially sit next to me, and may or may not approach the visitors**	yes	0.685	no	Removed from dataset.
**My cat will groom him/herself when I arrive home**	yes	0.648	no	Removed from dataset.
**I think my cat would be just as happy living with someone else (like the next door neighbour)**	yes	0.722	no	Remained in dataset. Used for analysis.
**My cat likes all people equally**	yes	0.545	no	Removed from dataset.
**My cat seems to know exactly how I feel and acts accordingly**	yes	0.420	no	Removed from dataset.
**I can never tell how my cat is going to react when I approach him/her**	no	0.050	no	Removed from dataset.
**If my cat was anxious, people other than me, might be just as good at comforting my cat**	yes	0.601	no	Removed from dataset.
**My cat appears aloof**	yes	0.646	no	Removed from dataset.

**My cat is clingy. I can't even go to the toilet in peace.**	yes	0.895	no	Remained in dataset. Used for analysis.
Subsection balance (items retained/total items in Subsection 2)				***11/30***
**Final balance (items remained/total items in Part 3 of the questionnaire)**				**26/93**

Table S4 - The twenty six reliable items retained for analysis

Item	Scale							
	Strongly disagree	Mainly disagree	Partly disagree	Neu-tral/ mixed	Partly agree	Mainly agree	Strongly agree	Not appli-cable
I don't know what I would do without my cat.								
My cat is my best friend.								
I think of my cat as like a child.								
I am very protective of my cat.								
I worry too much about my cat.								
If I were to tell my cat off and he/she would start purring or meowing at me, I would stop and try to make up with him/her.								
I am comfortable with being emotionally distant from my cat.								
I consider my cat part of the family.								
I talk to my cat every day.								
My cat and I often seem to work as a team that are in tune with each other.								
I don't think it is necessary to play with my cat regularly.								
When I go away my cat will play with and enjoy whoever is looking after him/her.								
My cat tends to approach visitors with his/her tail up and rub him/herself on their legs.								
My cat visits the neighbours (even if I am at home).								
I think my cat would be just as happy living with someone else (like the next door neighbour).								
My cat constantly follows me around the house.								
My cat is clingy. I can't even go to the toilet in peace.								
If I am in a separate room with a closed door, my cat will cry until I open the door.								
When I am about to leave the house, my cat will cry and try to leave with me.								
My cat will not eat if I go away.								
When worried or afraid, my cat. will seek me.								
My cat will often lick my hands or face.								
When my cat is eating, I like to stay with him/her until he/she has finished.								
I feel my cat doesn't like me as much as like him/her.								
My cat will never sit on my lap.								
When siting on my lap, being fussed, my cat's tail will sometimes thrash.								

Figure S1 - Median PC scores for groups A, B and C.

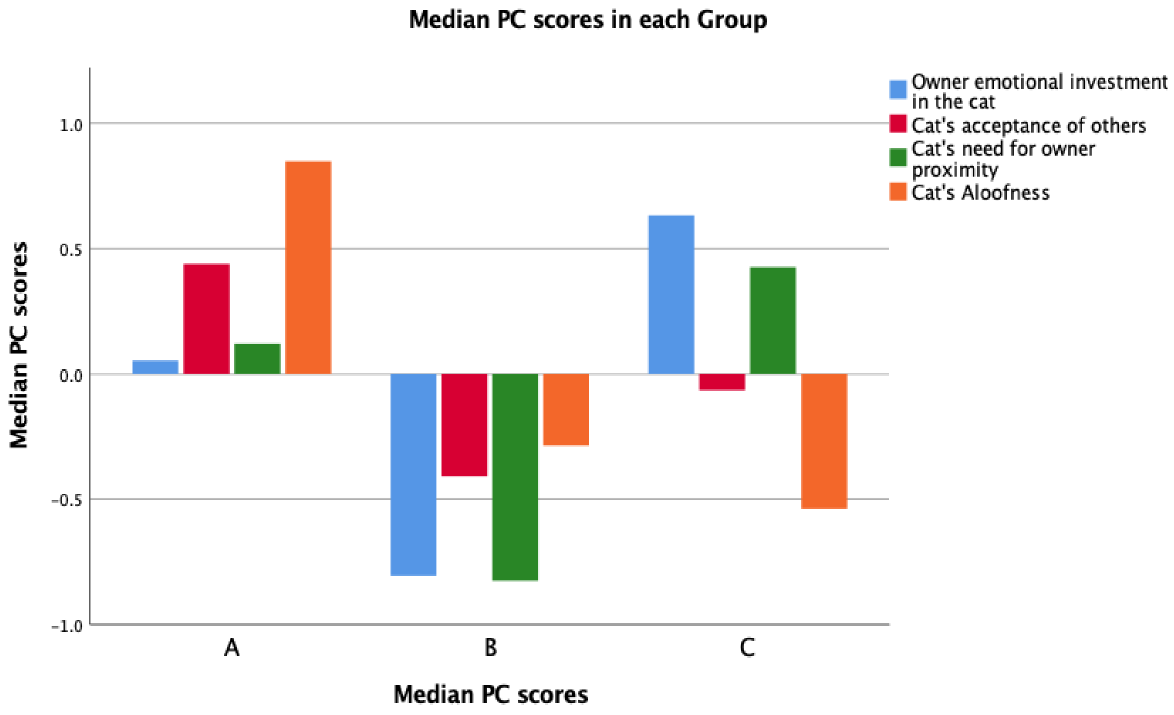

Table S5 - Principal Component Analysis, total variance explained by all components. All retained components had an eigenvalue greater than 1, (up to 5 component solution possible, based on the Kaiser criterion). The scree plot (S6) was then used to confirm whether a 4 or 5 factor solution should be used.

Total Variance Explained							
Component	Initial Eigenvalues			Extraction Sums of Squared Loadings			Rotation Sums of Squared Loadings
	Total	% of Variance	Cumulative %	Total	% of Variance	Cumulative %	Total
1	5.091	19.582	19.582	5.091	19.582	19.582	4.232
2	1.920	7.386	26.968	1.920	7.386	26.968	1.856
3	1.682	6.468	33.436	1.682	6.468	33.436	3.292
4	1.575	6.059	39.495	1.575	6.059	39.495	2.277
5	1.167	4.487	43.982	1.167	4.487	43.982	1.375
6	.974	3.746	47.728				
7	.950	3.653	51.381				
8	.927	3.564	54.945				
9	.887	3.410	58.355				
10	.868	3.338	61.693				
11	.857	3.297	64.990				
12	.804	3.092	68.082				
13	.781	3.002	71.084				
14	.731	2.810	73.894				
15	.713	2.742	76.636				
16	.695	2.673	79.309				
17	.688	2.647	81.956				
18	.643	2.471	84.428				
19	.624	2.398	86.826				
20	.593	2.280	89.107				
21	.572	2.200	91.307				
22	.531	2.041	93.348				
23	.506	1.948	95.295				
24	.493	1.898	97.194				
25	.436	1.676	98.869				
26	.294	1.131	100.000				

Figure S2 - Scree plot of PCA of relationship items, 4 factors chosen before the inflexion point.

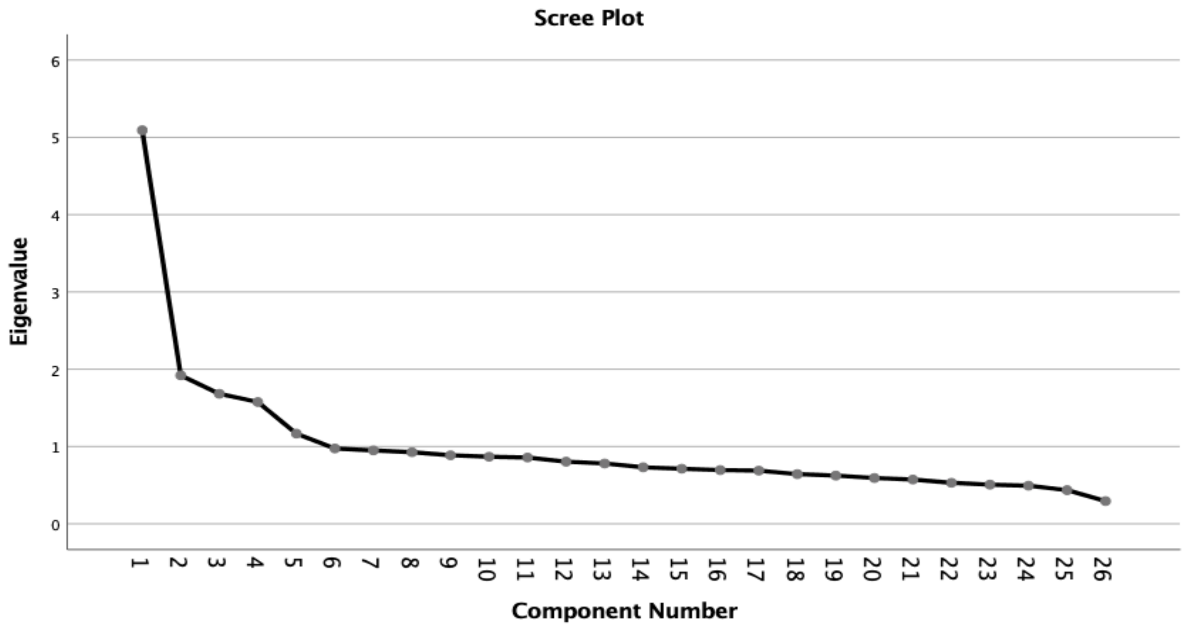

Table S6 - The median PC score for the groups A, B and C.

	"owner's emotional investment in the cat"	"cat's acceptance of others"	"cat's need for owner proximity"	"cat's aloofness"
Group A	0.05	0.44	0.12	0.85
Group B	-0.81	-0.41	- 0.83	-0.29
Group C	0.63	-0.06	0.43	-0.54

Table S7 - The median PC score for the groups A, B1, B2, C1 and C2.

	"owner's emotional investment in the cat"	"cat's acceptance of others"	"cat's need for owner proximity"	"cat's aloofness"
Group A	0.05	0.44	0.12	0.85
Group B1	-0.33	-1.02	- 0.60	-0.24
Group B2	-1.34	0.71	-0.98	-0.32
Group C1	0.67	-0.81	0.94	-0.18
Group C2	0.60	0.40	0	-0.71

Figure S3 – Box plot representing the score for Groups A, B1, B2, C1 and C2 for the item: “I don’t think it is necessary to play with my cat regularly”.

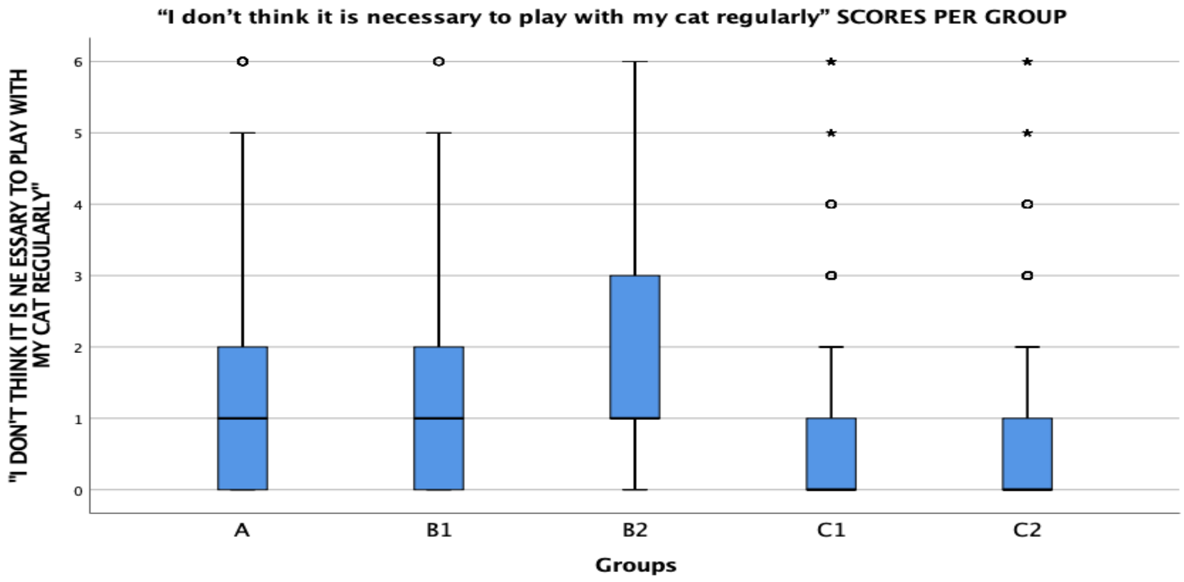

Figure S4 – Box plot representing the score for Groups A, B1, B2, C1 and C2 for the item: - “When worried or afraid, my cat will seek me”.

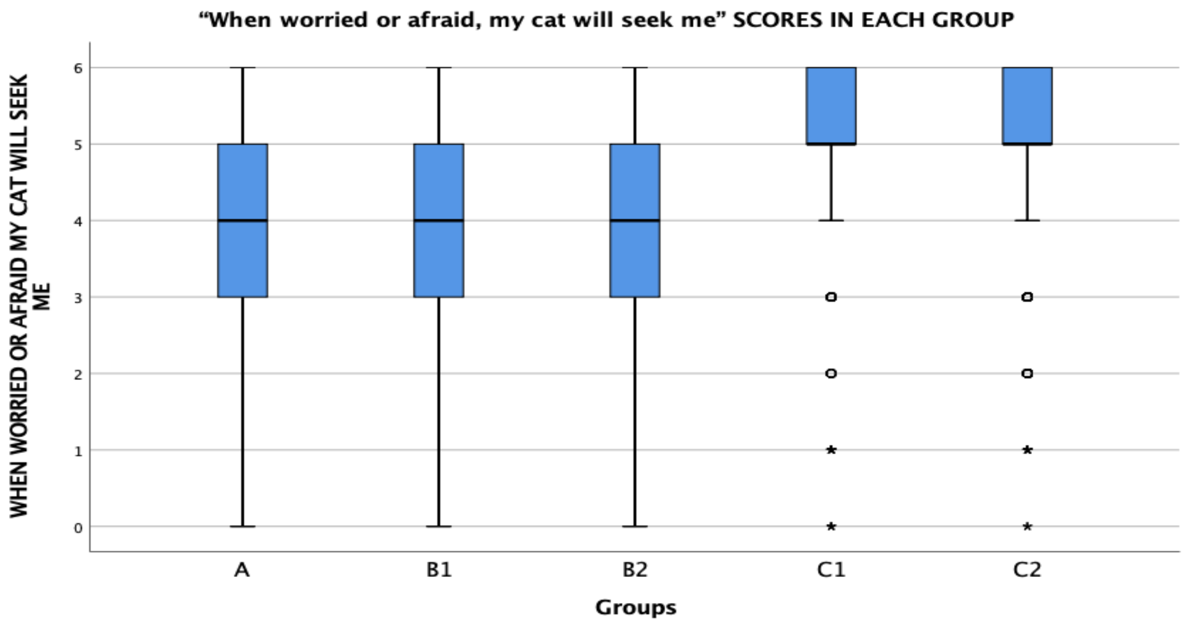

Figure S5 – Box plot representing the score for Groups A, B1, B2, C1 and C2 for the item: “My cat will often lick my hands or face”.

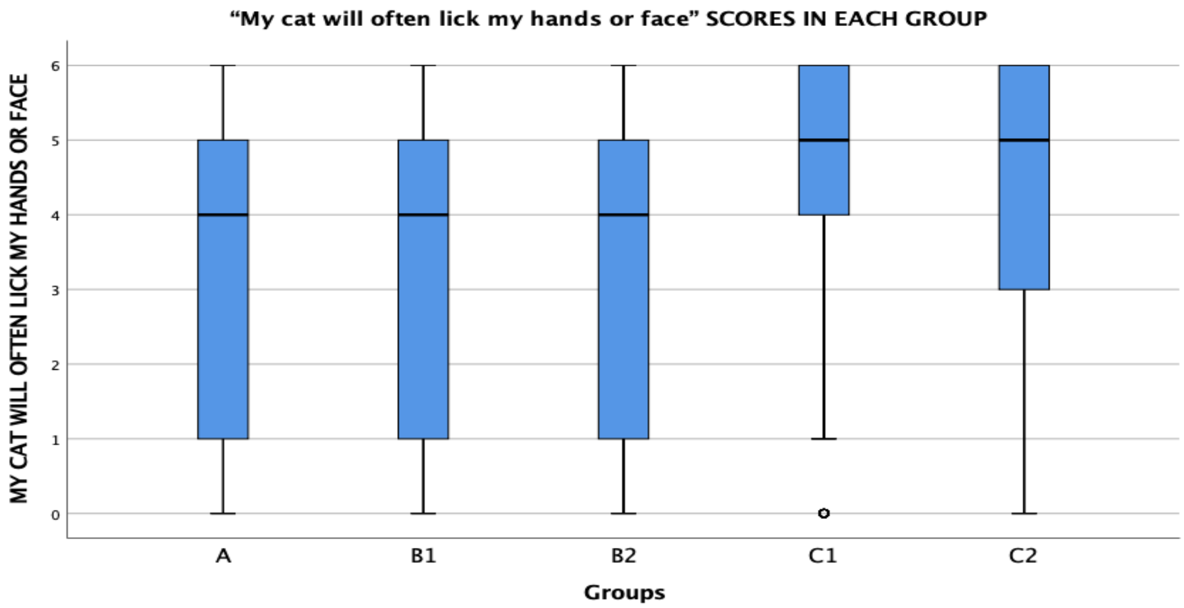

Figure S6 – Box plot representing the score for Groups A, B1, B2, C1 and C2 for the item: “When my cat is eating, I like to stay with him/her until he/she has finished”.

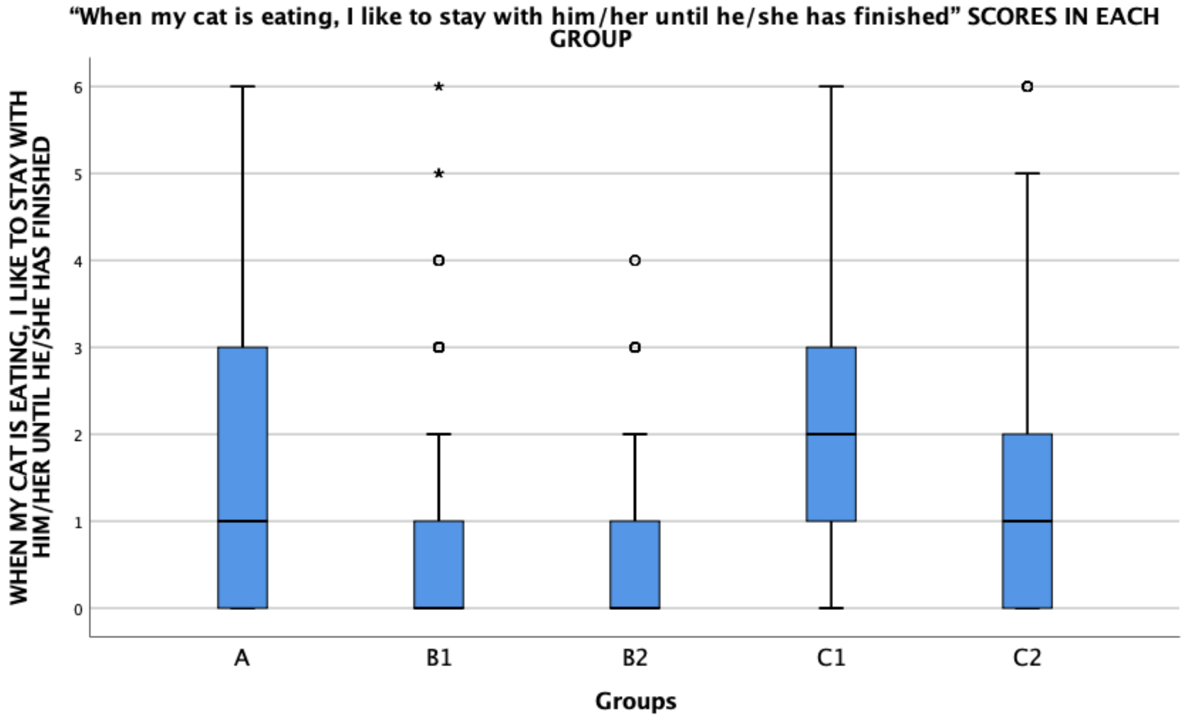

Figure S7 – Box plot representing the owner age distribution in Groups A, B1, B2, C1 and C2; mean +/- SD given on x-axis labels.

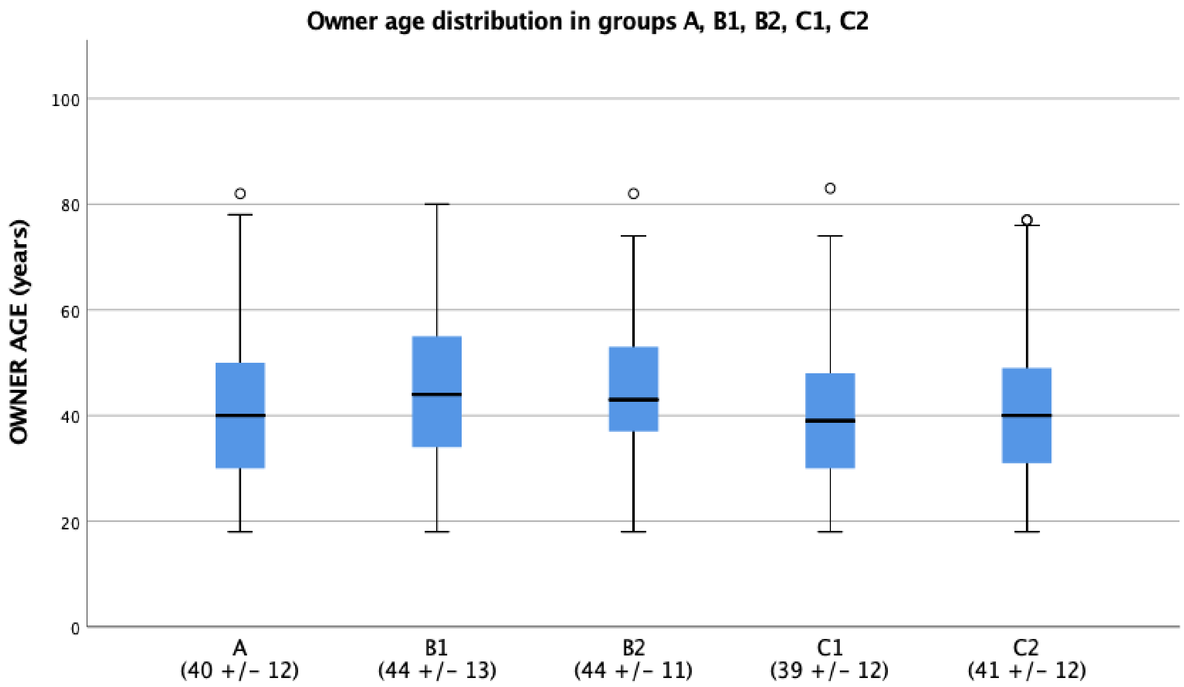

Figure S8 – Box plot representing the cat age distribution in Groups A, B1, B2, C1 and C2; mean +/- SD given on x-axis labels.

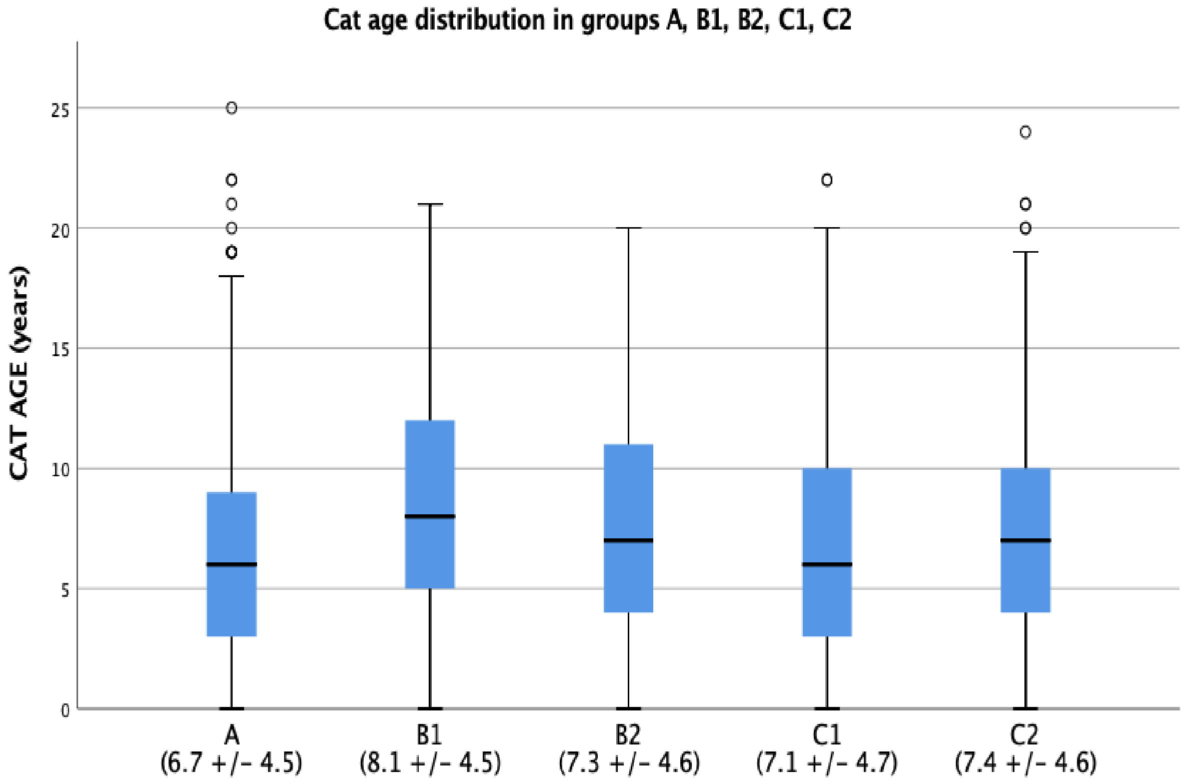

Information S1: English and Portuguese versions of final instruments and their relationship to the clusters identified in simple language terms.

ME and MY CAT – a rough guide: for a more detailed guide please read the full paper:

Please read the following statements and indicate how much you agree or disagree with each statement in relation to one cat you know well, by choosing one of the following statements: strongly agree, mainly agree, partly agree, neutral/mixed, partly disagree, mainly disagree, strongly disagree.

If the situation described in the statement hasn't occurred or been encountered by you or your cat, give your best estimate as to what you think would happen in that situation, but if you are really unsure, just ignore the question.

There are 22 statements.

Item		Response
1	I don’t know what I would do without my cat.	
2	My cat is my best friend.	
3	I think of my cat as like a child.	
4	I am very protective of my cat.	
5	I worry too much about my cat.	
6	If I were to tell my cat off and he/she would start purring or meowing at me, I would stop and try to make up with him/her.	
7	I am comfortable with being emotionally distant from my cat.	
8	I consider my cat part of the family.	
9	I talk to my cat every day.	
10	My cat and I often seem to work as a team that are in tune with each other.	
11	When I go away my cat will play with and enjoy whoever is looking after him/her.	
12	My cat tends to approach visitors with his/her tail up and rub him/herself on their legs.	
13	My cat visits the neighbours (even if I am at home).	
14	I think my cat would be just as happy living with someone else (like the next door neighbour).	
15	My cat constantly follows me around the house.	
16	My cat is clingy. I can’t even go to the toilet in peace.	
17	If I am in a separate room with a closed door, my cat will cry until I open the door.	
18	When I am about to leave the house, my cat will cry and try to leave with me.	
19	My cat will not eat if I go away.	
20	I feel my cat doesn’t like me as much as like him/her.	
21	My cat will never sit on my lap.	
22	When siting on my lap, being fussed, my cat’s tail will sometimes thrash.	

Once you have answered the above, to find out the type of relationship you and your cat have with each other follow the flow- diagram below, the questions relate to different coloured blocks of question above. Look at these and see how you tend to respond. In this diagram “mostly agree” means you agree at some level with most of the items in that block, likewise with “mostly disagree”. More details about each type of relationship are given afterwards.

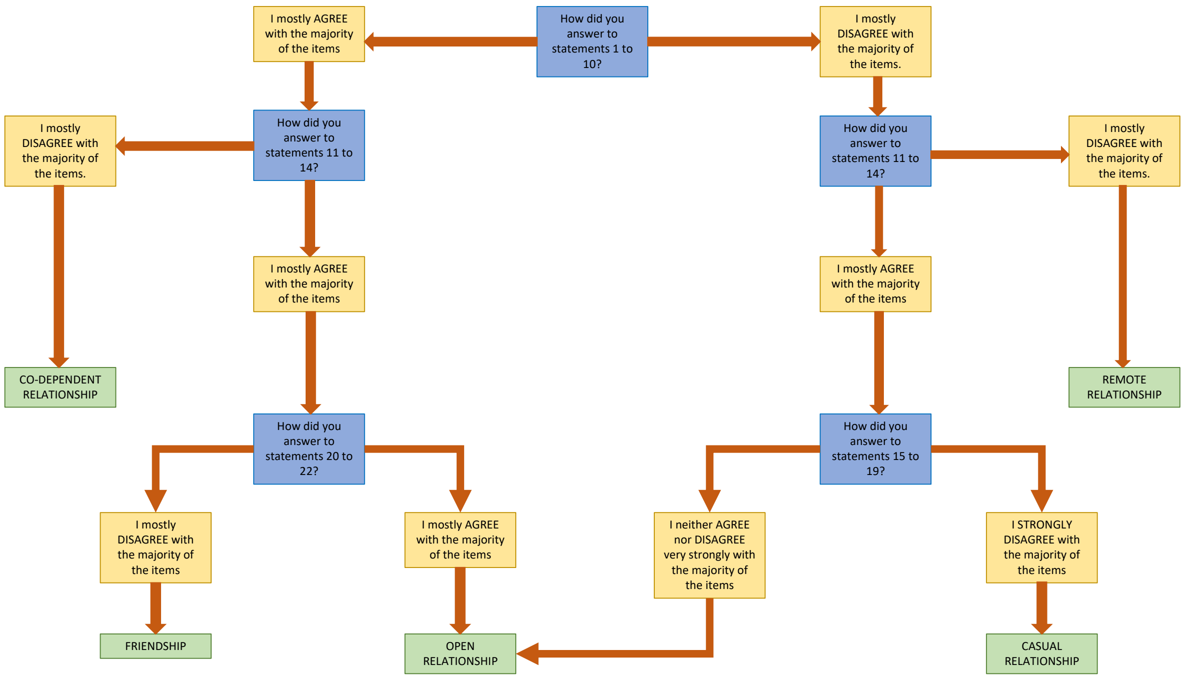

<u>THE CO-DEPENDENT RELATIONSHIP</u>

This cat has often come to depend on a very emotionally invested owner (the cat is very important to the owner, possibly seen as family or as a great friend).

The owner typically plays regularly with the cat, and is seen as a part of the same social group (the cat behaves in a friendly way towards the owner, even regularly licking the owner's hands and face) and as a secure base (the cat will seek the owner when worried).

The cat doesn't relate well with others (is even likely to hide when, for example, someone comes to the house).

This cat has learned that good things come when the owner is near (for example: the owner stays with the cat whilst the cat is eating) and so, makes a considerable effort to maintain physical proximity to the owner (these cats can even be considered to be clingy) and separation can be problematic (the cat may not eat if the owner is away). This might suggest the cat has some problems with controlling its frustrations.

This relationship is common among cats living in a one-person household with no access outdoors.

<u>THE FRIENDSHIP</u>

The owner is emotionally invested in the cat (worries about the cat, sees him/her as a good friend or a part of the family) and will often find time to play with the cat; the cat is very warm and friendly towards the owner (will often sit on the owner's lap), who is seen not only as part of the same social group (the cat will regularly lick the owner's hands and face) but also as a secure base (someone to seek out for comfort when the cat is worried).

The cat relates well to others (will greet or inspect visitors coming to the house and possibly even visit some neighbours). Alongside the friendly and warm relationship, cat and owner can happily function independently. This cat likes to be near the owner but doesn't feel a need to maintain physical proximity to the owner (doesn't always follow the owner around the house and may even take him/herself away to a preferred location) and separation is not likely to be problematic.

This relationship seems to occur more often in busy households with more than one cat, and the cats often have some outside access.

<u>OPEN RELATIONSHIP</u>

The cats in this type of bond reflect a prototypical view of the cat as a solitary, independent animal, who should have access to the outside. The cats relate well to other people (they are likely to greet visitors or visit neighbours) and have some affiliation with the owner, but have little need for owner proximity and may be seen as aloof (possibly will not sit on the owner's lap or lick the owner's hands and face).

REMOTE RELATIONSHIP

Cats in this type of relationship are cared for, but not typically considered to be a close friend or part of the family; they may not be very close emotionally, even though the cat's behaviour towards the owner may be quite friendly.

These cats prefer to maintain distance from people (owners and others), possibly because they lack confidence. They will not lick the owner's hands and face, nor will they seek the owner even when worried.

CASUAL RELATIONSHIP

These cats have always preferred life outdoors to life in their busy households. They will often visit several homes in their territory (possibly having more than one home!) and might be gone for days at the time.

Even though these cats behave in a friendly way towards the owner they will not try to stay close to their carer (in fact, the chance are they will even make an effort to put some distance between them and their owner).

EU e o MEU GATO – Um guia aproximado: para uma versão mais detalhada por favor consulte o artigo completo.

Por favor leia as seguintes afirmações e indique o quanto você concorda ou discorda de cada afirmação em relação a um gato que você conheça bem.
Para cada afirmação deverá escolher uma das seguintes opções: concordo completamente, concordo bastante, concordo um pouco, neutro, discordo um pouco, discordo bastante, discordo completamente.

Se a situação descrita na afirmação nunca ocorreu, ou nunca foi enfrentada por si ou pelo seu gato, por favor dê a sua melhor opinião sobre o que você acha que aconteceria nessa situação. Se você realmente não tiver certeza, selecione a opção "não se aplica".

Há 22 afirmações.

Afirmação		Resposta
1	Não sei o que faria sem o meu gato.	
2	O meu gato é o meu melhor amigo.	
3	Eu penso no meu gato como uma criança	
4	Sou muito protetor do meu gato.	
5	Eu preocupo-me demasiado com o meu gato.	
6	Se eu estivesse a ralhar com o meu gato e ele começasse a ronronar ou miar, eu parava de ralhar e tentava fazer as pazes com ele.	
7	Eu estou confortável em estar emocionalmente distante do meu gato.	
8	Eu considero o meu gato parte da família.	
9	Eu falo com o meu gato todos os dias.	
10	Eu e o meu gato somos uma equipa e estamos em sintonia um com o outro.	
11	Se eu for de férias o meu gato brinca e fica bem com quem estiver cuidando dele.	
12	Se tiver visitas, o meu gato geralmente aproxima-se com a cauda levantada e esfrega-se nas suas pernas.	
13	O meu gato visita os vizinhos (mesmo que eu esteja em casa).	
14	Eu penso que o meu gato seria igualmente feliz a viver com outra pessoa (como os vizinhos do lado).	
15	O meu gato segue-me constantemente pela casa.	
16	O meu gato é pegajoso. Nem consigo ir à casa de banho em paz.	
17	Se eu estiver num quarto com a porta fechada, o meu gato chora até eu abrir a porta.	
18	Quando eu estou prestes a sair de casa o meu gato chora e tenta sair comigo.	
19	O meu gato não come se eu for de férias.	
20	Eu sinto que o meu gato não gosta de mim tanto quanto eu gosto dele/a.	
21	O meu gato nunca se senta no meu colo.	
22	Quando o meu gato está no meu colo e lhe estou a tocar, por vezes ele abana a cauda.	

Depois de ter respondido ao questionário, para descobrir o tipo de relacionamento que existe entre você e o seu gato, siga o fluxograma abaixo. As perguntas no fluxograma estão relacionadas com grupos de afirmações, assinaladas com cores diferentes no questionário. Olhe para as afirmações em cada grupo e veja como tende a responder. Neste diagrama, "concordo maioritariamente" significa que você concorda em algum nível com a maioria das afirmações naquele grupo, da mesma forma que "discordo maioritariamente" significa que você discorda em algum nível com a maioria das afirmações naquele grupo.
Mais detalhes sobre cada tipo de relacionamento são fornecidos abaixo.

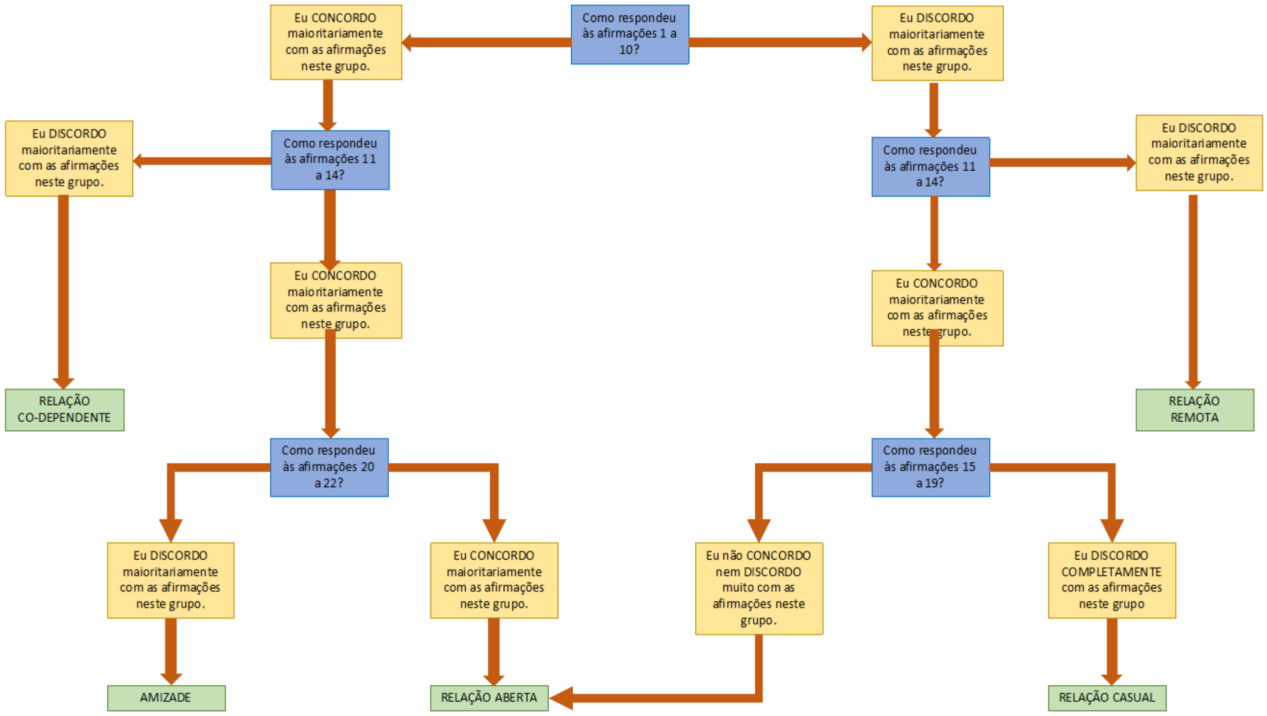

RELAÇÃO CO-DEPENDENTE

Este gato habitou-se a depender de um dono muito emocionalmente investido (o gato é muito importante para o dono, possivelmente é considerado como parte da família ou um grande amigo).
O dono brinca regularmente com o gato que é visto como parte do mesmo grupo social (o gato tem um comportamento amigável para com o dono, lambendo-lhe regularmente as mãos e por vezes, o rosto) e como um porto seguro (quando está preocupado o gato procura o dono).
O gato não se relaciona bem com outras pessoas (é até provável que se esconda quando, por exemplo, há visitas).
Este gato aprendeu que as coisas boas acontecem quando o dono está por perto (por exemplo: o dono fica com o gato enquanto ele come) então faz um esforço considerável para se manter próximo do dono (podendo até ser considerado pegajoso). A separação pode ser problemática (o gato pode não comer se o dono estiver ausente) o que pode sugerir que o gato tem alguns problemas para controlar as suas frustrações.
Esta relação é comum em gatos que vivem em casa de uma só pessoa, sem acesso ao exterior.

A AMIZADE

O dono está emocionalmente investido no gato (preocupa-se com o gato, vê-o como um bom amigo ou como parte da família) e procura brincar com ele; o gato comporta-se de forma calorosa e amigável com o dono (senta-se muitas vezes no seu colo), que é visto não apenas como parte do mesmo grupo social (o gato regularmente lambe as mãos e o rosto do dono), mas também como um porto seguro (alguém onde encontrar conforto quando preocupado).
O gato relaciona-se bem com outras pessoas (cumprimenta ou inspeciona pessoas que venham a casa e possivelmente até visita alguns vizinhos).
Apesar de terem uma relação amigável e calorosa, o gato e o dono podem funcionar independentemente. Este gato gosta de estar perto do dono, mas não sente a necessidade de manter proximidade física com o dono (nem sempre segue o dono pela casa e pode até retirar-se para um local preferido) e é provável que a separação não seja problemática.
Essa relação parece ocorrer com mais frequência em famílias onde existe mais de um gato, e estes gatos geralmente têm algum tipo de acesso ao exterior.

RELAÇÃO ABERTA

Os gatos neste tipo de relação refletem a visão prototípica do gato como um animal solitário e independente, que deveria ter acesso ao exterior. Estes gatos relacionam-se bem com outras pessoas (são propensos a cumprimentar visitantes ou

visitar vizinhos) e têm alguma afiliação com o dono, mas têm pouca necessidade de proximidade com ele e podem ser vistos como indiferentes (possivelmente não se vão sentar no colo do dono ou lamber-lhe as mãos ou o rosto).

RELAÇÃO REMOTA

Os gatos, neste tipo de relacionamento, são cuidados, mas normalmente não são considerados amigos próximos ou parte da família e mesmo que o comportamento do gato em relação ao dono seja bastante amigável, isso não se traduz, necessariamente, em proximidade emocional.

Estes gatos preferem manter distância das pessoas (donos e outros), possivelmente por falta de confiança. Os gatos não vão lamber as mãos ou o rosto do dono e, mesmo quando preocupados, não o vão procurar.

RELAÇÃO CASUAL

Estes gatos preferem a vida ao ar livre e podem até estar fora de casa por vários dias de cada vez. Eles costumam visitar várias casas no seu território (possivelmente tendo mais do que um lar!)

Mesmo que estes gatos se comportem de maneira amigável com o seu dono, não é provável que tentem ficar perto dele (na verdade, eles podem até mesmo fazer um esforço para manter alguma distância entre eles e os seus donos).
